# Integrative Single‐Cell and Spatial Transcriptomics Reveal Organelle Stress–Associated Heterogeneity and Immune Microenvironment Remodeling in Lung Adenocarcinoma

**DOI:** 10.1155/humu/4471260

**Published:** 2026-05-13

**Authors:** Xiaoyan Chang, Meifeng Li, Chenghao Wang, Xiang Zhou, Yupeng Zhao, Simiao Chen, Linyou Zhang

**Affiliations:** ^1^ Department of Thoracic Surgery, Second Affiliated Hospital of Harbin Medical University, Harbin, China, hrbmush.edu.cn; ^2^ Department of Immunology, Harbin Medical University, Harbin, China, hrbmu.edu.cn

**Keywords:** immunotherapy, multiomics, organelle stress, SLC16A14, tumor microenvironment

## Abstract

**Background:**

Lung adenocarcinoma (LUAD) exhibits marked heterogeneity. Organelle stress–adaptive programs that tumor cells develop under hypoxia, nutrient limitation, and proteostasis pressure may drive functional reprogramming of tumor biology, remodel the immune microenvironment, and ultimately influence the benefits of immunotherapy. Therefore, it is necessary to systematically characterize the coordinated changes across organelle stress–related pathways in LUAD, establish a subtyping and prognostic stratification framework, and identify key molecules as well as potential cell–cell communication axes.

**Methods:**

Transcriptomic profiles and clinical follow‐up data from The Cancer Genome Atlas LUAD (TCGA‐LUAD) cohort and public cohorts were collected. Pathway activities were quantified using organelle stress–related gene sets, and core stress programs associated with overall survival (OS) and progression‐free survival (PFS) were screened by Cox regression and Kaplan–Meier analyses. Nonnegative matrix factorization (NMF) was used for unsupervised subtyping and stability evaluation. Functional enrichment, genomic features, and immune landscapes were compared between subtypes, and potential benefit from immune checkpoint blockade (ICB) was inferred using tumor immune dysfunction and exclusion (TIDE) and immunophenoscore (IPS). Single‐cell RNA sequencing (scRNA‐seq) and spatial transcriptomics (ST) were integrated to characterize malignant cell states, spatial niches, and cell–cell communication networks. In LUAD cell lines, the candidate gene was silenced by small interfering RNA (siRNA), and phenotypic assays were performed to validate its effects.

**Results:**

The organelle stress activity‐based system robustly classified LUAD into two biologically distinct subtypes (the mitochondrial–ribosome biogenesis, MRB, subtype and the lysosomal catabolism, LC, subtype), which showed systematic differences in prognosis and functional programs. The MRB subtype exhibited enhanced stress and metabolic adaptation accompanied by immune exclusion features, whereas the LC subtype showed a relatively immune‐active tumor microenvironment. Immunotherapy‐related analyses suggested divergent trends in potential ICB benefit between the two subtypes. Multiscale evidence highlighted SLC16A14 (MCT14) as a key node linking stress heterogeneity to malignant progression. At the single‐cell level, SLC16A14 was mainly expressed in malignant cells, and communication analyses suggested that CALCR‐related signaling may mediate tumor–endothelial interactions and contribute to an immune‐excluded microenvironment. In vitro, SLC16A14 silencing suppressed tumor cell proliferation, invasion, and migration, supporting its role as a key molecule connecting stress adaptation and tumor progression.

**Conclusion:**

We established an organelle stress program‐based subtyping and prognostic framework for LUAD, revealed the coupling between stress adaptation and TME remodeling, and proposed SLC16A14 and its associated communication network as potential intervention targets, providing multiomics evidence for interpreting LUAD heterogeneity and for stratifying immunotherapy and combination strategies.

## 1. Introduction

Lung cancer remains one of the leading causes of cancer‐related mortality worldwide, and lung adenocarcinoma (LUAD) is the most common histological subtype of non–small cell lung cancer, with a high incidence and substantial disease burden that varies across populations and regions [1–3]. The major clinical challenge of LUAD lies not only in its invasive and metastatic potential and risk of recurrence but also in its pronounced heterogeneity [4–7]. LUAD displays marked interpatient and intratumoral heterogeneity at both histological and molecular levels [2, 6, 8]. Large‐scale cohorts and multiregion sequencing studies further suggest that tumor evolution and subclonal competition shape tumor plasticity and therapeutic tolerance, resulting in substantial divergence in prognosis and treatment benefit even among patients with the same stage or similar driver backgrounds [4, 6, 7]. Although targeted therapies and immune checkpoint blockade (ICB) have reshaped the treatment landscape for advanced LUAD, overall response rates and durable benefits remain limited, and treatment benefit varies widely among individuals [9–13]. Therefore, it is critical to identify new frameworks that can explain heterogeneity and support patient stratification and treatment optimization from the perspective of intrinsic tumor adaptive programs and tumor microenvironment (TME) remodeling [5, 11, 14].

During tumor initiation and progression, cancer cells are chronically exposed to adverse conditions such as hypoxia, nutrient deprivation, oxidative stress, proteostasis burden, and therapeutic pressure; thus, stress adaptation has become a core biological feature of tumors [5, 15]. At the organelle level, endoplasmic reticulum stress, mitochondrial homeostasis imbalance, ribosome regulation, and lysosome–autophagy remodeling jointly support tumor adaptation and survival [16–20]. Importantly, tumor cells can also reshape the TME in a non–cell‐autonomous manner [5, 14]. For example, sustained endoplasmic reticulum stress not only promotes tumor cell survival, angiogenesis, and metastatic tolerance but may also drive immunosuppressive networks and weaken antitumor immune surveillance [18, 21]. The integrated stress response (ISR), a translation reprogramming pathway, can couple selective translation with transcriptional programs to maintain key adaptive phenotypes under pressure and has been linked to immunotherapy responses [16, 17, 20, 22]. In addition, autophagy can be induced by stress in both tumor cells and immune cells; its impact on antigen presentation, inflammatory signaling, and immune cell function is highly context‐dependent, making it a double‐edged sword in immune evasion and therapy sensitivity [19, 23]. Beyond classical stress pathways, neuropeptide‐like factors secreted by tumor and stromal cells may also participate in microenvironment shaping [24, 25]. For instance, adrenomedullin (ADM) signaling is closely related to tumor angiogenesis, and calcitonin gene–related peptide (CGRP; encoded by CALCA) can modulate innate immune responses, suggesting that neuroimmune–vascular axes may contribute to stress adaptation and remodeling of the TME [25–29].

We focused on the link between organelle stress adaptation and TME remodeling in LUAD. We systematically profiled the transcriptomic landscape of organelle stress–related pathways and established a robust stress‐adaptive subtyping system for prognostic stratification and biological interpretation. By characterizing coordinated changes in organelle stress pathways, we identified two subtypes with distinct biological meanings and clinical outcomes: one characterized by enhanced metabolic and organelle homeostasis remodeling, increased genomic instability, and immune exclusion features, and the other exhibited a relatively immune‐active TME. In addition, using immune phenotyping metrics and immunotherapy response prediction scores, we observed divergent trends in potential ICB benefit across stress subtypes. We further identified SLC16A14 as a key molecule contributing to subtype differences; it is mainly expressed in tumor cells and is associated with worse prognosis in LUAD. SLC16A14 correlated with multiple tumor‐related pathways, and its knockdown attenuated proliferation, invasion, and migration in vitro. Cell–cell communication analyses suggested that SLC16A14+ tumor cells may communicate with endothelial cells through CALCR‐related signaling, involving ligand–receptor pairs such as ADM/CALCA and CALCRL, potentially contributing to vascular remodeling and an immune‐excluded microenvironment.

## 2. Materials and Methods

### 2.1. Data Acquisition

Bulk data of the LUAD cohort from The Cancer Genome Atlas (TCGA), including transcriptomic, genomic, and clinical information, were downloaded from the Genomic Data Commons (GDC) Data Portal (https://portal.gdc.cancer.gov/). Transcriptomic data were obtained as RNA‐seq gene expression quantification in transcripts per million (TPM), and genomic data were obtained as masked somatic mutation files for single‐nucleotide variants. Clinical information was primarily retrieved from the GDC Data Portal and further complemented by clinical phenotypes for the same samples from cBioPortal [30] (https://www.cbioportal.org/), and then merged into a unified clinical dataset for downstream survival and subtyping analyses.

The LUAD scRNA‐seq dataset GSE127465 [31] was obtained from the Tumor Immune Single‐cell Hub (TISCH) database [32]. We downloaded the standardized expression matrix and accompanying metadata generated by TISCH and used the cell‐type annotations provided by TISCH in all downstream single‐cell analyses. The 10× Visium‐based spatial transcriptomic dataset was obtained from the BioStudies database [33] (https://www.ebi.ac.uk/biostudies/, Accession Number: E‐MTAB‐13530), and sample P17_T1 was included in this study.

Organelle stress–related gene sets were downloaded from the Molecular Signatures Database (MSigDB [34]), and details are provided in Supporting Information 2: Table S1. Single‐sample gene set enrichment analysis (ssGSEA) was performed using the R package GSVA [35] to calculate pathway activity scores, followed by normalization to generate a sample‐level organelle stress pathway activity matrix for downstream analyses. Immune cell infiltration was quantified by ssGSEA using previously published marker gene sets for 28 immune cell types, derived from the immunogenomic framework reported by Charoentong et al. [36].

### 2.2. Single‐Cell and Spatial Transcriptomic Data Processing

For scRNA‐seq, we followed the unified processing workflow provided by TISCH, which applies a standardized pipeline to perform quality control, batch effect handling, clustering, and cell‐type annotation. Cell‐type annotations and malignant status labels were directly adopted from the TISCH metadata, and downstream analyses were performed using Seurat in R [37].

Spatial transcriptomic data were analyzed using Seurat for 10× Visium data. All spots in the selected sample were included. Normalization was performed using SCTransform. Principal component analysis (PCA) was used for dimensionality reduction, and the Top 15 principal components were used for clustering (resolution = 0.8). Spatial niches were annotated based on marker gene expression (EPCAM for tumor regions, LYZ and DCN for immune/stromal regions, and SFTPC for normal regions). MCP‐counter was used to infer immune and stromal cell signatures at the spot level to support and validate niche annotation.

### 2.3. Screening of Prognosis‐Related Organelle Stress Pathways and Survival Analyses

In the TCGA‐LUAD cohort, univariate Cox regression analyses were performed using ssGSEA scores of each organelle stress pathway as predictors for overall survival (OS) and progression‐free survival (PFS), respectively, to identify pathways significantly associated with prognosis. Pathways that were significant in both OS and PFS analyses were intersected and defined as the LUAD‐specific prognosis‐related organelle stress pathway set. For Kaplan–Meier analyses, the optimal cutoff for high and low pathway activity groups was determined using maximally selected rank statistics, and differences between groups were evaluated using the log‐rank test.

### 2.4. NMF‐Based Organelle Stress Subtyping and Stability Evaluation

Based on the pathway activity matrix of the prognosis‐related organelle stress pathway set, unsupervised decomposition and consensus clustering were conducted using the R package NMF with the Brunet algorithm. The factorization rank was surveyed from *k* = 2 to 10, with 10 repeated runs to assess clustering stability, and a fixed random seed (seed = 123,456) was used to ensure reproducibility. Cophenetic correlation, silhouette, residuals, and RSS were jointly considered to determine the optimal number of subtypes. Two robust subtypes were obtained in TCGA‐LUAD and named according to their core stress features. The subtype system was subsequently used for systematic comparisons of molecular, genomic, and immune microenvironment characteristics.

### 2.5. Differential Expression Analysis, Functional Enrichment, and Key Gene Identification

To characterize transcriptomic differences between subtypes, differential expression analysis was performed on the TPM expression matrix using limma [38], with |log_2_ fold change| and false discovery rate (FDR) as thresholds to define differentially expressed genes (DEGs). Functional enrichment analysis was conducted using the R package clusterProfiler [39] to perform Gene Ontology (GO) enrichment for DEGs, identifying systematic biological differences between subtypes. Key genes associated with stress subtypes were screened by intersecting subtype‐upregulated DEGs, prognosis‐related genes for OS and PFS, and tumor‐versus‐normal DEGs, followed by integrative evaluation of expression patterns, prognostic relevance, immune microenvironment associations, and malignant cell–specific expression in scRNA‐seq. SLC16A14 was prioritized as the core candidate gene for downstream mechanistic inference and in vitro validation.

### 2.6. Genomic Instability and Somatic Mutation Landscape

Somatic mutation data were obtained from TCGA‐LUAD SNV annotations and analyzed using maftools [40] for visualization and statistical comparison, including oncoplots and comparisons of mutation frequencies between subtypes. Genomic instability‐related features were collected from two sources: Tumor mutational burden (TMB) and microsatellite instability (MSI) were retrieved from cBioPortal, while homologous recombination deficiency (HRD) and aneuploidy scores were obtained from the TCGA pancancer immunogenomic dataset described in The Immune Landscape of Cancer by Thorsson et al. [14]. After aligning with subtype labels at the sample level, these indicators were compared between subtypes.

### 2.7. Immune Microenvironment Characterization and Immunotherapy Response Prediction

At the bulk level, the ESTIMATE R package [41] was used to calculate ImmuneScore and StromalScore and to infer tumor purity, enabling assessment of immune/stromal composition across subtypes. Immune cell infiltration was quantified by ssGSEA using gene signatures for 28 immune cell types, and immune function–related gene sets were further scored to describe the immune landscape from both cellular composition and functional states. Immunotherapy‐related phenotypes included differential expression of immune checkpoints (ICPs) and immunogenic cell death (ICD)–related genes. Potential benefit from ICB was inferred using the TIDE framework [22], focusing on T‐cell dysfunction and immune exclusion scores. The dysfunction score reflects the degree of T‐cell functional impairment in inflamed tumors, whereas the exclusion score reflects the extent to which immune cells are prevented from infiltrating the tumor parenchyma; higher scores generally indicate more prominent immune evasion and a lower likelihood of response to ICB. For spatial transcriptomics, MCP‐counter [42] was applied to deconvolve immune and stromal signatures at the spot level, followed by correlation analyses and spatial visualization with pathway activities or key gene expression to validate immune exclusion phenotypes in tissue space.

### 2.8. Cell–Cell Communication Analyses in Single‐Cell and Spatial Settings

CellChat [43] was used to infer cell–cell communication networks in scRNA‐seq based on ligand–receptor interactions, with aggregation and comparison at the signaling pathway level. Within malignant cells, subgroups were defined according to SLC16A14 expression status to characterize communication outputs of SLC16A14+ malignant cells. Analyses focused on overall interaction number and strength, outgoing and incoming communication patterns, communication modes, and cell roles within specific pathways. Based on these results, the CALCR signaling axis and its key components involved in tumor–endothelial interactions were examined.

To validate single‐cell‐inferred communication at the spatial scale, the R package NICHES was used to model and quantify ligand–receptor interactions at the spot level in spatial transcriptomics, enabling estimation of interaction strength across different spatial niches. Interaction signals were mapped back to tissue space based on niche annotations, and key axes consistent with CellChat results (e.g., CALCR‐related signaling) were evaluated for interaction strength and spatial enrichment patterns.

### 2.9. Cell Culture and Small Interfering RNA (siRNA) Transfection

Human LUAD cell lines A549 and H1299 were used for in vitro validation. A549 cells were cultured in F‐12K medium, and H1299 cells were cultured in RPMI‐1640 medium, both supplemented with 10% fetal bovine serum and 1% penicillin/streptomycin at 37°C with 5% CO_2_. siRNA targeting SLC16A14 was transiently transfected; siRNA sequences are provided in Supporting Information 3: Table S2. Lipofectamine RNAiMAX was used for transfection at a final concentration of 50 nM, and knockdown efficiency was evaluated by western blot at 48 h posttransfection.

### 2.10. Western Blotting

Total protein was extracted using RIPA lysis buffer and quantified by the bicinchoninic acid (BCA) method. Equal amounts of protein (30 *μ*g per lane) were separated by SDS‐PAGE and transferred onto membranes. Membranes were blocked with nonfat milk for 90 min at room temperature and incubated with primary antibodies at 4°C overnight (GAPDH, 1:2000, CST, Cat. 2118; MCT14, 1:2000, Proteintech, Cat. 26953‐1‐AP). After washing with TBST, membranes were incubated with HRP‐conjugated antirabbit secondary antibody (CST, Cat. 7074), and signals were detected using ECL reagents.

### 2.11. Tumor Cell Phenotypic Assays

Cell proliferation was assessed using the Cell Counting Kit‐8 (CCK‐8) assay at 0, 24, 48, and 72 h. Cell migration was evaluated using a wound‐healing assay; images were captured at 0 and 24 h after scratching, and the wound closure rate was calculated. Cell invasion was assessed using a Transwell assay with Matrigel‐coated inserts; invading cells were fixed, imaged, and counted at 24 h. All experiments were performed with at least three independent biological replicates, and imaging, quantification, and statistical analyses were conducted using consistent criteria.

### 2.12. Statistical Analysis

All statistical analyses were mainly performed in R (Version 4.1.2). For comparisons between two groups, Student′s *t*‐test or the Wilcoxon rank‐sum test was applied according to data distribution. For multiple group comparisons, one‐way ANOVA or the Kruskal–Wallis test was used with multiple testing correction when appropriate. Categorical variables were compared using the chi‐square test or Fisher′s exact test. Survival analyses were performed using Cox regression and the log‐rank test. Correlations were assessed using Spearman′s method. A two‐sided *p* value < 0.05 was considered statistically significant.

## 3. Results

### 3.1. Univariate Cox Regression Identifies Prognosis‐Related Organelle Stress Pathways and K‐M Validation

**Figure 1:**
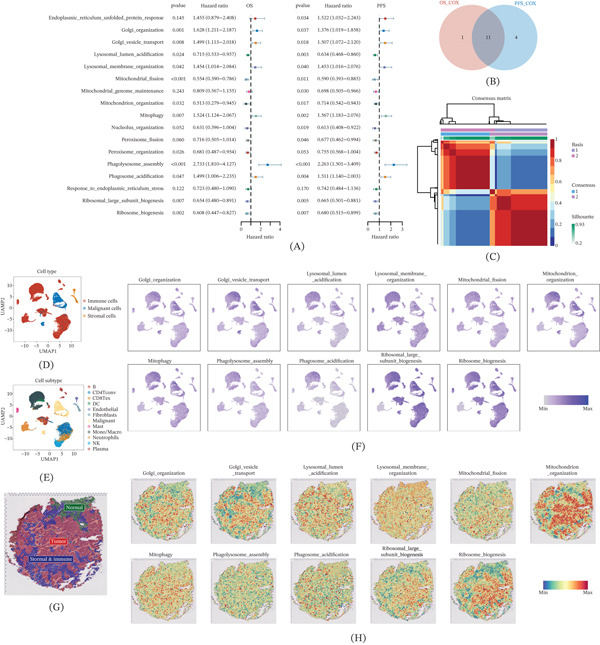
Identification of prognosis‐related organelle stress pathways in LUAD. (A) Univariate Cox regression based on organelle stress pathway scores in the TCGA‐LUAD cohort to evaluate associations with OS and PFS. (B) Intersection of pathways significantly associated with both OS and PFS. (C) Consensus matrix of NMF clustering based on the intersected prognosis‐related pathway scores, indicating two robust subtypes. (D) UMAP visualization of major cell types in the scRNA‐seq dataset. (E) UMAP visualization of annotated subclusters. (F) Single‐cell distribution of representative prognosis‐related pathway activities. (G) Niche annotation of the spatial transcriptomic section (tumor, stromal and immune, normal). (H) Spatial distribution of representative pathway activities; colors indicate normalized pathway activity.

We first quantified the activity of organelle stress–related pathways in TCGA‐LUAD using GSVA‐derived ssGSEA scores and performed univariate Cox regression analyses for OS and PFS to screen prognosis‐related organelle stress programs (Figure 1A). Pathways that were significant in both OS and PFS analyses were intersected as LUAD‐specific prognosis‐related organelle stress pathways (Figure 1B), yielding 11 pathways including Golgi_organization, Golgi_vesicle_transport, and lysosomal_lumen_acidification. Each pathway in the intersected set was further evaluated by Kaplan–Meier analyses, and all pathways significantly stratified OS and PFS between high‐activity and low‐activity groups, supporting the robustness of this pathway set for prognostic stratification (Supporting Information 1: Figure S1).

### 3.2. NMF Identifies Two Organelle Stress Subtypes

Based on the prognosis‐related organelle stress pathway set, we performed NMF to define stress subtypes. The factorization rank was surveyed from *k* = 2 to 10 with 100 repeated runs under a fixed random seed (seed = 123,456), and *k* = 2 provided the optimal balance between stability and model fit, supported by cophenetic, silhouette, residuals, and RSS metrics (Supporting Information 1: Figure S2), and the consensus matrix further suggested robust two‐class clustering (Figure 1C). To localize the subtyping pathways within tumor ecosystems, we integrated scRNA‐seq and spatial transcriptomics. In scRNA‐seq, cell‐type (Figure 1D) and subcluster (Figure 1E) annotations from TISCH were adopted, and multiple stress pathways such as Golgi_organization and Golgi_vesicle_transport were preferentially enriched in malignant cells (Figure 1F). In spatial transcriptomics, after niche annotation (Supporting Information 1: Figure S3A–D, Figure 1G), most pathways such as mitochondrion_organization and ribosome_biogenesis showed higher activity in tumor niches (Figure 1H), whereas some pathways (e.g., lysosomal_membrane_organization and phagosome_acidification) were distributed across both tumor and peritumoral niches.

### 3.3. Distinct Stress Features and Functional Enrichment Differences Between Subtypes

**Figure 2:**
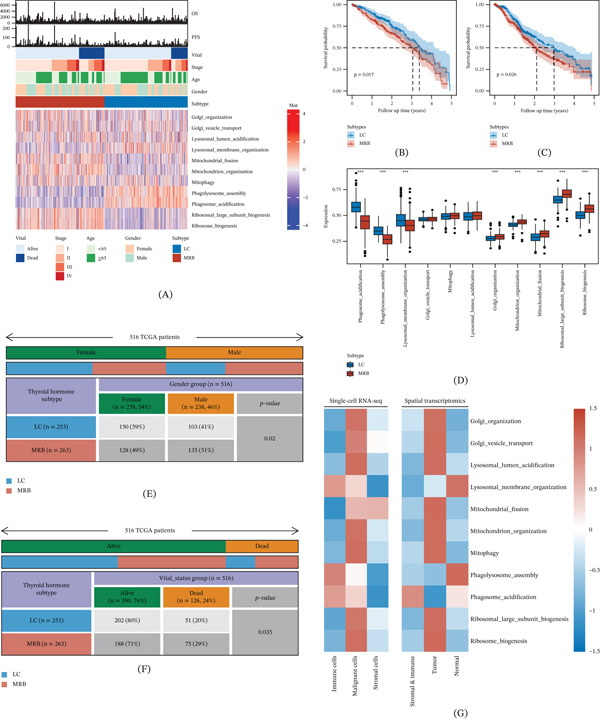
Associations between organelle stress subtypes and clinical phenotypes. (A) Heatmap of prognosis‐related organelle stress pathway scores with clinical annotations and subtype labels. (B) Kaplan–Meier curves showing worse OS in the MRB subtype compared with the LC subtype. (C) Kaplan–Meier curves showing worse PFS in the MRB subtype compared with the LC subtype. (D) Comparison of prognosis‐related pathway scores between subtypes. ∗∗∗*p* < 0.001. (E) Association between subtypes and sex. (F) Association between subtypes and survival status. (G) Consistent subtype‐associated pathway activity patterns across single‐cell components (immune, malignant, stromal) and spatial niches (stromal and immune, tumor, normal).

We summarized the two subtypes and corresponding clinical phenotypes using a heatmap (Figure 2A). The subtypes displayed distinct stress pathway activity profiles (Figure 2D). The subtype characterized by ribosome‐ and mitochondrion‐related stress pathways was defined as the MRB (mitochondrial–ribosome biogenesis) subtype, whereas the subtype characterized by phagosome‐ and lysosome‐related pathways was defined as the LC (lysosomal catabolism) subtype. MRB and LC showed significant differences in OS (Figure 2B) and PFS (Figure 2C). In terms of clinical characteristics, the MRB subtype included more male patients and fewer female patients than the LC subtype (Figure 2E), and the OS rate was markedly lower in the MRB subtype (Figure 2F). MRB signature pathways were mainly enriched in malignant cells and tumor regions in both single‐cell and spatial analyses, whereas LC signature pathways were more enriched in immune cells and localized to peritumoral niches (Figure 2G).

**Figure 3:**
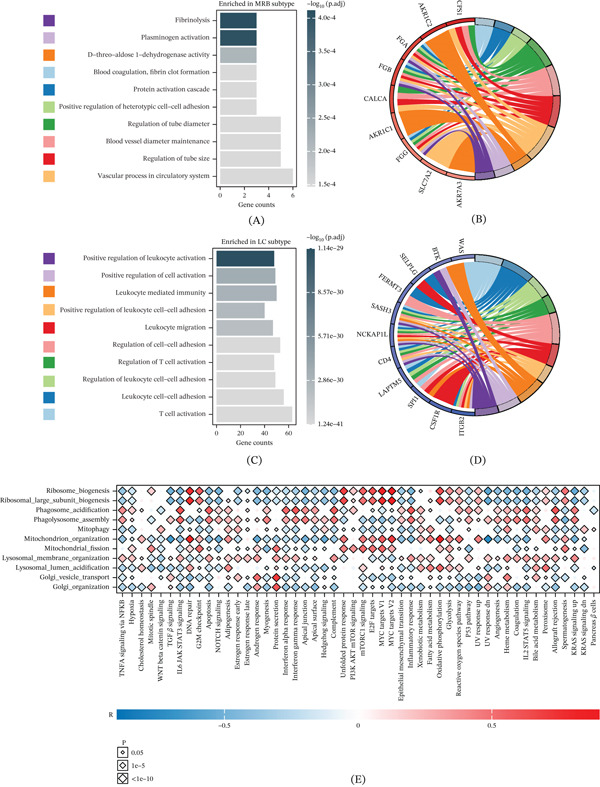
Functional divergence of LC and MRB subtypes and links to oncogenic programs. (A) GO enrichment of DEGs upregulated in the MRB subtype, highlighting coagulation, fibrinolysis, and vascular‐related processes. (B) Associations between representative MRB genes and enriched functional categories. (C) GO enrichment of DEGs upregulated in the LC subtype, highlighting leukocyte activation, immune adhesion/migration, and T‐cell activation. (D) Associations between representative LC genes and immune‐related functional categories. (E) Correlation between prognosis‐related organelle stress pathway activities and Hallmark gene set activities; color indicates correlation direction and magnitude, and symbol size indicates significance.

Functional enrichment analysis of subtype‐specific genes showed that the MRB subtype was preferentially enriched in coagulation‐ and vascular remodeling–related processes, including fibrinolysis, plasminogen activation, and regulation of vascular caliber (Figure 3A,B). In contrast, the LC subtype was more strongly enriched in immune activation and leukocyte migration processes, such as T‐cell activation, leukocyte migration, and leukocyte‐mediated immunity (Figure 3C,D). We further evaluated the association between organelle stress pathway activities and Hallmark gene set activities and observed structured correlations between stress programs and oncogenic pathways. MRB‐associated pathways were correlated with proliferation, cell cycle progression, and metabolic reprogramming (e.g., MYC targets, E2F/G2M, mTORC1 signaling, oxidative phosphorylation, and glycolysis), whereas LC‐associated lysosomal/phagosome‐related pathways were significantly linked to immune‐related programs (e.g., interferon responses and complement) (Figure 3E). These results suggest that the organelle stress–based subtyping system captures heterogeneous states of oncogenic programs and TME in LUAD.

### 3.4. The MRB Subtype Exhibits Stronger Genomic Instability and Mutation Burden

**Figure 4:**
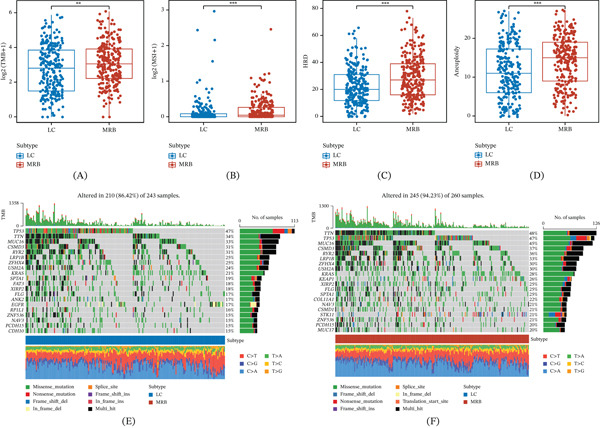
Distinct genomic landscapes between LC and MRB subtypes. (A–D) Comparisons of (A) TMB, (B) MSI, (C) HRD, and (D) aneuploidy between subtypes. ∗∗*p* < 0.01, ∗∗∗*p* < 0.001. (E) Oncoplot for the LC subtype showing frequently mutated genes, sample‐level mutation burden, and mutation type composition. (F) Oncoplot for the MRB subtype showing frequently mutated genes, sample‐level mutation burden, and mutation type composition.

Given the pronounced functional divergence between MRB and LC, we compared their genomic characteristics. The MRB subtype showed significantly higher levels of TMB, MSI, HRD, and aneuploidy (Figure 4A–D) and exhibited an overall higher frequency of somatic alterations in the mutation landscape compared with the LC subtype (Figure 4E,F). These results indicate that the stress state of the MRB subtype is accompanied by broader genomic instability, which may enhance intrinsic tumor adaptation while shaping an unfavorable TME through mutation‐ and aneuploidy‐related effects.

### 3.5. Comprehensive Immune Microenvironment Analyses of the Two Subtypes

**Figure 5:**
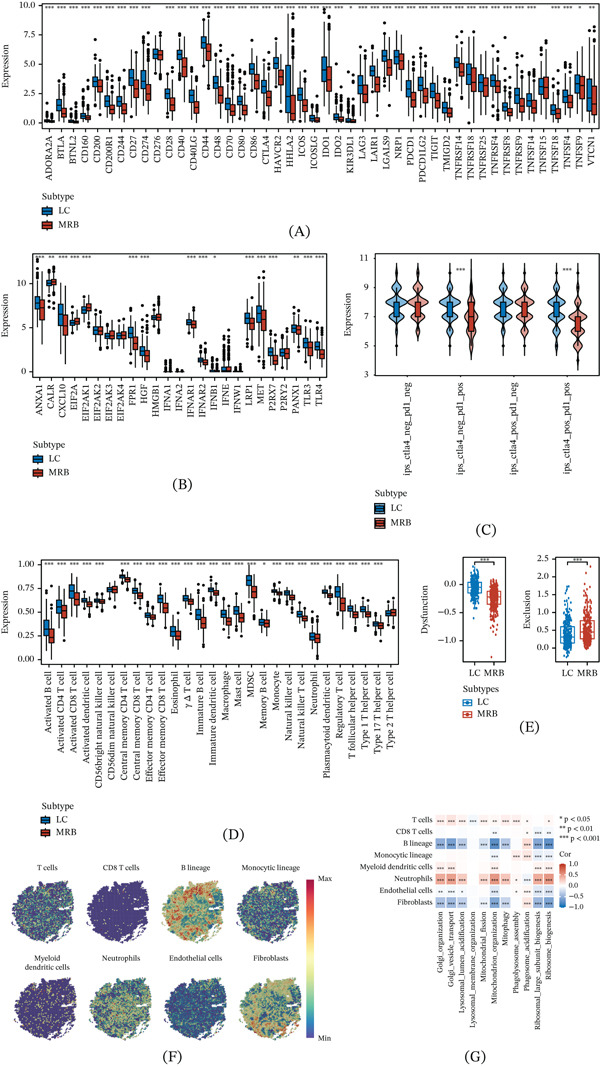
Immune microenvironment differences between LC and MRB subtypes. (A) Differential expression of immune checkpoint (ICP) genes between subtypes. (B) Differential expression of immunogenic cell death (ICD)–related genes between subtypes. (C) Comparisons of immunophenoscore (IPS) under different anti‐CTLA‐4 and anti‐PD‐1 response scenarios. (D) Differences in ssGSEA‐based infiltration scores for 28 immune cell types between subtypes. (E) Differences in TIDE‐derived T‐cell dysfunction and immune exclusion scores between subtypes. (F) Spatial distribution of representative MCP‐counter deconvolved signatures. (G) Correlation heatmap between prognosis‐related pathway activities and MCP‐counter scores. ∗*p* < 0.05, ∗∗*p* < 0.01, ∗∗∗*p* < 0.001.

We systematically evaluated immune microenvironment differences between the two subtypes. At the transcriptomic level, ICP genes and ICD‐related genes were generally higher in the LC subtype (Figure 5A,B), consistent with its more immune‐active context. In immunotherapy‐related analyses, the LC subtype showed higher IPS scores in ctla4_neg_pd1_pos and ctla4_pos_pd1_pos scenarios (Figure 5C) and broadly higher infiltration scores for multiple immune cell types in the 28‐cell ssGSEA analysis (Figure 5D). In contrast, TIDE analysis indicated stronger immune exclusion in the MRB subtype, whereas the dysfunction score did not show a concordant increase; this may be due to the overall low immune infiltration in an immune‐excluded context, which provides insufficient signal to elevate absolute dysfunction scores (Figure 5E).

Deconvolution results further suggested that the MRB subtype was associated with lower overall antitumor immune infiltration (e.g., CD8 T cells, activated CD4 T cells, and NK cells) and displayed a subtype‐biased immune composition (Figure 5D). At the spatial level, the MCP‐counter was used to infer the spatial distribution of immune and microenvironmental cell signatures (Figure 5F). Correlation analyses between organelle stress pathway activity and spatial MCP‐counter scores showed that LC‐associated pathways such as phagolysosome assembly and phagosome acidification exhibited spatial patterns consistent with antitumor immune cells (T cells and B cells), whereas MRB‐associated mitochondrion‐ and ribosome‐related pathways colocalized with neutrophil signatures and were negatively correlated with B cell and stromal signatures (endothelial cells and fibroblasts) (Figure 5G). These findings support distinct immune states of the TME between the two stress subtypes.

### 3.6. Identification of the Core Gene SLC16A14 and In Vitro Phenotypic Validation

**Figure 6:**
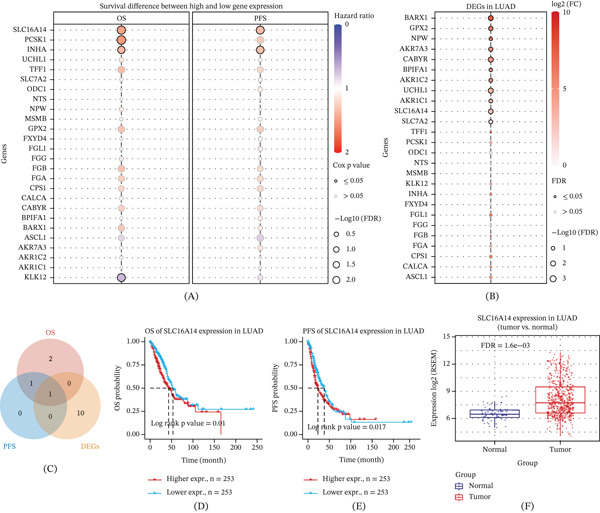
Identification of SLC16A14 as a key gene associated with organelle stress patterns. (A) Bubble plot summarizing survival associations of MRB‐upregulated candidate genes with OS and PFS; color indicates hazard ratio, and bubble size indicates significance. (B) Overview of differential expression of candidate genes between LUAD tumors and normal tissues. (C) Venn diagram showing the overlap among OS‐related genes, PFS‐related genes, and tumor‐versus‐normal DEGs, identifying SLC16A14 as the shared candidate gene. (D) Kaplan–Meier curve for OS comparing high versus low SLC16A14 expression groups. (E) Kaplan–Meier curve for PFS comparing high versus low SLC16A14 expression groups. (F) Expression difference of SLC16A14 between tumor and normal tissues.

To identify potential intervention targets linking organelle stress to TME remodeling, we intersected MRB‐upregulated DEGs with OS‐ and PFS‐associated prognostic genes and LUAD tumor‐versus‐normal DEGs and identified SLC16A14 as a key candidate gene (Figure 6A–C). High SLC16A14 expression was significantly associated with worse OS and PFS (Figure 6D,E) and was markedly upregulated in LUAD tumors compared with normal tissues (Figure 6F).

**Figure 7:**
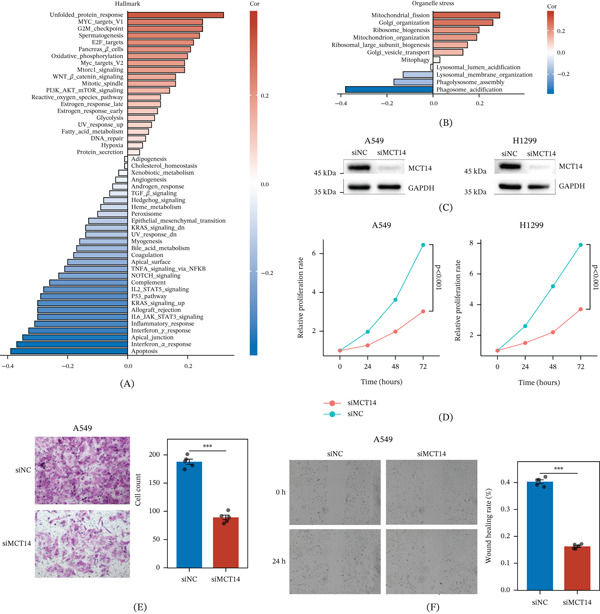
Association of SLC16A14 with malignant phenotypes in LUAD. (A) Correlation analysis between SLC16A14 expression and Hallmark gene set activities. (B) Correlation analysis between SLC16A14 expression and organelle stress pathway activities. (C) Western blot validation of MCT14 knockdown in A549 and H1299 cells. (D) Proliferation curves showing reduced growth after MCT14 knockdown. (E) Transwell invasion assay showing decreased invasion after MCT14 knockdown. ∗∗∗*p* < 0.001. (F) Wound‐healing assay showing reduced migration after MCT14 knockdown.

In Hallmark pathway analyses, SLC16A14 expression was positively correlated with mitosis‐related programs (e.g., MYC targets, G2M_checkpoint, E2F_targets, and mitotic_spindle) and metabolic reprogramming pathways (e.g., mTORC1_signaling, glycolysis, and fatty_acid_metabolism) and negatively correlated with immune activation programs (e.g., interferon_alpha_response, interferon_gamma_response, and complement), supporting its association with LUAD progression and an immunosuppressive microenvironment (Figure 7A). Consistently, SLC16A14 showed significant positive correlations with MRB signature pathways and negative correlations with LC signature pathways, indicating that it captures intrinsic program differences between the two stress subtypes (Figure 7B).

In vitro, knockdown of MCT14 encoded by SLC16A14 was validated by western blot (Figure 7C) and significantly inhibited the proliferation of A549 and H1299 cells (Figure 7D). MCT14 knockdown also reduced invasion (Figure 7E) and migration (Figure 7F), supporting a promalignant role of SLC16A14 in LUAD.

### 3.7. Association of SLC16A14 With an Immunosuppressive Microenvironment in LUAD

**Figure 8:**
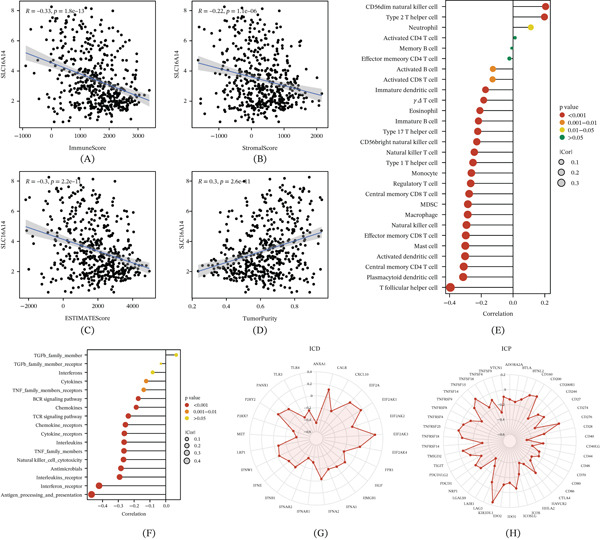
Associations between SLC16A14 and the immune microenvironment. (A–D) Correlations of SLC16A14 with (A) ImmuneScore, (B) StromalScore, (C) ESTIMATE score, and (D) tumor purity. (E) Correlations between SLC16A14 and immune cell infiltration scores; dot size indicates the absolute correlation coefficient, and color indicates significance. (F) Correlations between SLC16A14 and immune functional programs (e.g., antigen processing and presentation, interferon pathways, cytokine/chemokine‐related signaling, and TCR and BCR signaling). (G) Radar plot summarizing associations between SLC16A14 and ICD‐related features. (H) Radar plot summarizing associations between SLC16A14 and ICP‐related features.

To link SLC16A14 to an immunosuppressive TME, we systematically assessed its associations with microenvironmental metrics. In ESTIMATE analyses, SLC16A14 was negatively correlated with ImmuneScore, StromalScore, and the overall ESTIMATE score and positively correlated with tumor purity (Figure 8A–D), suggesting a broader association with an immune‐cold TME. Consistently, SLC16A14 was negatively correlated with multiple antitumor immune cell signatures (e.g., activated CD4 T cells, CD8 T cells, and NK cell–related components) (Figure 8E) and negatively associated with immune pathway modules such as antigen processing and presentation as well as cytokine/chemokine‐related signaling (Figure 8F). Radar plots summarized its overall relationships with ICD and ICP features, showing widespread negative associations between SLC16A14 and immune activation factors (Figure 8G,H).

### 3.8. Communication Analyses Suggest Characteristic CALCR Signaling From SLC16A14+ Malignant Cells to Endothelial Cells

**Figure 9:**
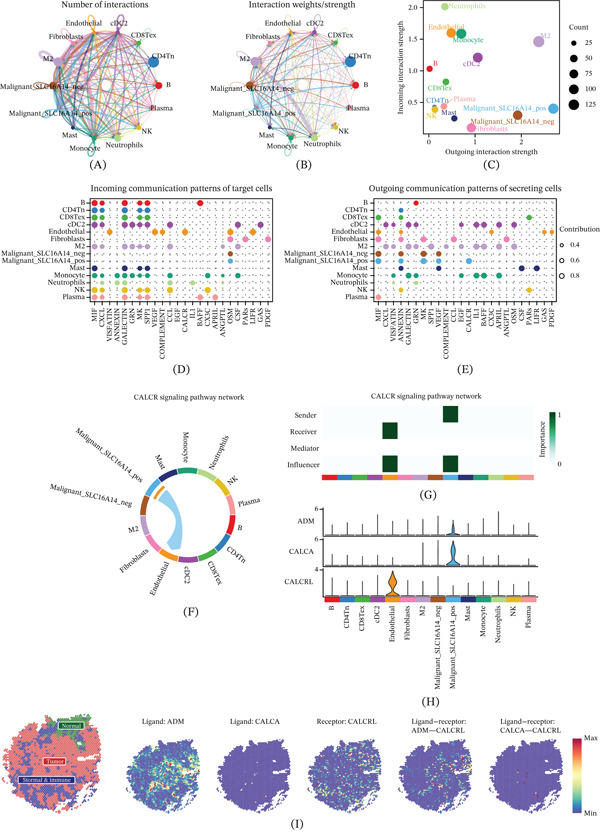
Cell–cell communication analysis of SLC16A14+ malignant cells. (A) Global communication network showing the number of interactions among cell populations. (B) Global communication network showing interaction strength among cell populations. (C) Bubble plot showing outgoing and incoming interaction strengths across cell populations. (D) Bubble plot of incoming signaling pathways for target cells. (E) Bubble plot of outgoing signaling pathways for target cells. (F) Chord diagram of the CALCR signaling pathway showing CALCR signals sent from SLC16A14+ malignant cells to endothelial cells. (G) Role analysis of different cell populations within the CALCR pathway. (H) Expression distribution of CALCR pathway‐related genes across cell populations. (I) Spatial visualization of CALCR pathway ligands, receptors, and interaction strength, supporting spatial consistency of tumor–endothelial signaling.

To further investigate the intercellular context of SLC16A14 in LUAD, we performed CellChat analyses to characterize communication patterns of SLC16A14+ malignant cells. Global interaction networks summarized the number and strength of interactions among major cell populations (Figure 9A,B). Outgoing and incoming strength analyses showed that SLC16A14+ malignant cells displayed the strongest outgoing signaling (Figure 9C), motivating pathway‐level dissection. Communication pattern analyses indicated that SLC16A14+ malignant cells specifically sent CALCR pathway signals and endothelial cells were the primary receivers of CALCR signaling (Figure 9D,E). Network visualization further supported that CALCR signaling was mainly emitted by SLC16A14+ malignant cells and targeted endothelial cells (Figure 9F). Role analysis suggested that SLC16A14+ malignant cells acted as senders and influencers, whereas endothelial cells acted as receivers and influencers in the CALCR pathway (Figure 9G). At the gene level, CALCA and ADM were prominently expressed in SLC16A14+ malignant cells, whereas CALCRL was more highly expressed in endothelial cells, supporting potential tumor‐to‐endothelium communication via CALCR‐related signaling (Figure 9H).

We further used spatial transcriptomics to validate the expression of CALCR pathway ligands and receptors and to quantify their interaction strength in tissue space (Figure 9I). Spatial analyses showed that CALCR pathway components were present at the tissue level, with ADM and CALCA enriched in tumor regions and CALCRL mainly enriched in immune/stromal regions. Ligand–receptor interactions were concentrated at the interface between tumor and immune/stromal regions, providing spatial support for the tumor–endothelial interactions inferred from scRNA‐seq and suggesting that CALCR‐related signaling participates in tumor‐driven regulation of endothelial function.

## 4. Discussion

LUAD resides in a highly heterogeneous TME ecosystem, and ICB yields durable benefit only in a subset of patients [8, 9, 12]. A central challenge is to move beyond single biomarkers to identify biological programs that reflect both intrinsic tumor states and interactions among TME components, in which stress adaptation plays a critical regulatory role [5, 18, 44]. Stress responses encompassing proteostasis, metabolic reprogramming, and organelle quality control are considered key features that support tumor survival under hypoxia, nutrient limitation, and immune pressure [45]. Meanwhile, immunology studies suggest that organelle stress and related signaling are not merely tumor cell–intrinsic events; they can reprogram myeloid and lymphoid cells toward tolerance and immunosuppression, linking intrinsic stress states with immune evasion [21, 44]. Based on this rationale, we propose that organelle stress programs may provide new targets for LUAD stratification and treatment strategies and help explain immune heterogeneity.

In this study, we quantified organelle stress pathways from transcriptomic data and integrated OS and PFS analyses to identify prognosis‐related organelle stress programs. Using NMF‐based unsupervised clustering, we defined two robust subtypes: the MRB subtype and the LC subtype. This is also in line with other recent molecular subtyping studies in LUAD [46]. The two subtypes differed markedly in clinical features, indicating that mitochondrion‐ and ribosome‐related stress pathways characteristic of MRB may contribute to the prognostic differences between subtypes. Cross‐scale analyses integrating single‐cell and spatial transcriptomics further showed that these pathways were preferentially enriched in malignant components and tumor niches, suggesting that tumor cells upregulate stress programs in response to microenvironmental challenges to support adaptation.

Biologically, MRB and LC represent two distinct stress‐adaptive modes accompanied by divergent immune microenvironment outcomes. MRB is defined by coordinated enhancement of mitochondrial organization and ribosome biogenesis, typically consistent with proliferative and biosynthetic states and synergistic with MYC/E2F‐driven cell cycle progression and mTORC1‐associated anabolic metabolism. This aligns with the logic that mitochondrial remodeling together with translational control can sustain tumor growth under stress by enhancing energy supply and biosynthesis, while increasing dependence on quality control and stress signaling. In contrast, LC is biased toward lysosome‐centered catabolic programs, often linked to autophagy–lysosome flux and antigen processing pathways, and in our study co‐occurred with immune activation and leukocyte migration features. Overall, LUAD may differentiate between an MRB‐like anabolic/proliferative mode and an LC‐like catabolic/immune‐interactive mode, with substantial differences in immune context and potential treatment responses.

A key finding is that the MRB subtype is accompanied by stronger genomic instability and a higher somatic alteration burden. Although higher mutation burden may increase potential neoantigen sources, accumulating evidence indicates that chromosomal instability and aneuploidy are often associated with immune evasion, reduced immune infiltration, and decreased immunotherapy response. In particular, tumor aneuploidy has been reported to correlate with immune evasion features and reduced benefit from immunotherapy [47], suggesting that copy number and aneuploidy‐driven states may offset the immunogenicity advantages conferred by point mutations. Within our framework, the higher aneuploidy and HRD features of the MRB subtype provide a plausible explanation for immune exclusion associated with stress adaptation: Genomic remodeling may enhance stress signaling and phenotypic plasticity, while weakening effective antitumor immunity through altered antigen presentation, interferon signaling, and microenvironment remodeling. Thus, the MRB subtype may represent a highly fit and plastic tumor state in which stress tolerance and genomic remodeling jointly promote an immunosuppressive TME.

Subtype differences also point to implications for immunotherapy. The LC subtype shows a more immune‐active background with higher ICP expression, often interpreted as an interferon‐driven negative feedback state, suggesting some degree of immune engagement. More broadly, inflammatory markers may also reflect disease progression in other settings [48]. In contrast, the MRB subtype is characterized by immune exclusion, where effector cells fail to enter or establish effective tumor contact. Our results support that MRB‐like LUAD may be less suitable for ICB monotherapy and may require combination strategies that simultaneously target intrinsic stress dependencies and relieve stromal/vascular barriers or myeloid suppression, whereas LC‐like tumors may have a more favorable immune context for ICB benefit.

We further propose SLC16A14 (encoding MCT14) as a core tumor‐intrinsic node linking organelle stress heterogeneity to malignant behavior. By integrating multiple evidence layers, we prioritized SLC16A14 as a candidate gene at the intersection of key pathways and validated its protumor effects on proliferation, migration, and invasion in vitro. Although MCT14 remains less characterized than classical lactate transporters in the SLC16 family, it is well recognized that monocarboxylate metabolism and acid‐base balance can profoundly influence tumor–immune interactions. Beyond intrinsic effects, communication analyses suggested that SLC16A14+ malignant cells preferentially signal to endothelial cells through CALCR‐related pathways. This ligand–receptor axis is immunologically relevant because endothelial cells are key gatekeepers for immune cell entry into tumors. Previous studies have shown that ADM can drive tumor angiogenesis and vascular remodeling through CALCRL receptor complexes, contributing to abnormal perfusion and barriers to immune infiltration. In addition, CALCA‐related neuropeptide signaling is increasingly recognized in peripheral immune regulation. Therefore, SLC16A14+ malignant cells may represent a stress‐adaptive phenotype with anabolic features that may help shape a vascular niche via CALCRL‐related signaling, consolidating immune exclusion and promoting progression.

This study has limitations. First, the subtype framework and immunotherapy‐related analyses were mainly derived from retrospective cohorts and should be validated in prospective LUAD cohorts with real‐world ICB treatment. Second, the direct molecular mechanisms by which SLC16A14 regulates stress adaptation and the immune microenvironment require further investigation. Third, the spatial transcriptomic analysis was based on a single representative section, which limited the evaluation of intersample spatial heterogeneity.

In summary, we established an MRB/LC subtyping system for LUAD based on organelle stress–related transcriptional programs and revealed systematic differences in prognosis, genomic instability, and immune microenvironment states. Multiomics integration suggested that the MRB subtype is characterized by enhanced stress and metabolic adaptation with immune exclusion features, whereas the LC subtype exhibits a relatively immune‐active ecosystem and distinct trends in predicted immunotherapy benefit. We further positioned SLC16A14 as a key node linking stress heterogeneity to malignant progression and propose that it may participate in tumor–endothelial interactions and immune exclusion through CALCR‐related signaling.

## Author Contributions

Xiaoyan Chang: conceptualization, data curation, formal analysis, methodology, visualization, and writing—original draft; Meifeng Li: investigation, methodology, and validation; Chenghao Wang: data curation, investigation, methodology, and validation; Xiang Zhou: investigation, resources, and validation; Yupeng Zhao: investigation, resources, and validation; Simiao Chen: data curation, methodology, visualization, and writing—review and editing; Linyou Zhang: conceptualization, project administration, supervision, and writing—review and editing.

## Funding

No funding was received for this manuscript.

## Disclosure

All authors read and approved the final manuscript.

## Ethics Statement

The authors have nothing to report.

## Conflicts of Interest

The authors declare no conflicts of interest.

## Supporting Information

Additional supporting information can be found online in the Supporting Information section.

## Supporting information


**Supporting Information 1** Figures S1–S3 include Kaplan–Meier survival validation of the intersected prognosis‐related pathways, determination of the optimal factorization rank for NMF subtyping, and spot annotation in spatial transcriptomics based on representative markers. Figure S1: Kaplan–Meier survival validation of the intersected prognosis‐related pathways. Pathways were dichotomized into high‐ and low‐activity groups, and OS and PFS were compared using Kaplan–Meier curves. Figure S2: Determination of the optimal factorization rank for NMF subtyping. Cophenetic, dispersion, evar, residuals, RSS, silhouette, and sparseness metrics were evaluated across ranks to determine the optimal value. Figure S3: Spot annotation in spatial transcriptomics based on representative markers. (A) H&E image of the tissue section. (B) Unsupervised clustering of spatial spots. (C) Spatial expression patterns of representative markers (tumor: EPCAM; stromal/immune: DCN, LYZ; normal: SFTPC) for niche annotation. (D) UMAP of spatial spots colored by niche type (tumor, stromal and immune, normal).

**Supporting Information 2** Table S1: Organelle stress–related gene sets.

**Supporting Information 3** Table S2: siRNA sequences.

## Data Availability

Publicly available datasets were analyzed in this study. TCGA‐LUAD transcriptomic, somatic mutation, and clinical data were downloaded from the Genomic Data Commons (GDC) Data Portal. Clinical annotations were additionally retrieved from cBioPortal. The LUAD scRNA‐seq dataset is available under accession GSE127465 (accessed via the TISCH database). The 10× Visium spatial transcriptomic dataset is available in BioStudies under accession E‐MTAB‐13530 (Sample P17_T1). The organelle stress–related gene sets were obtained from MSigDB.
