# Supplementary material for: Integrative Single‐Cell and Spatial Transcriptomics Reveal Organelle Stress–Associated Heterogeneity and Immune Microenvironment Remodeling in Lung Adenocarcinoma
Source: Hum Mutat. 2026 May 13;2026:4471260. doi: 10.1155/humu/4471260 (PMC13168938; doi:10.1155/humu/4471260)
Supplement: Supplementary file 1 — Supporting Information 1 Figures S1–S3 include Kaplan–Meier survival validation of the intersected prognosis‐related pathways, determination of the optimal factorization rank for NMF subtyping, and spot annotation in spatial transcriptomics based on representative markers. Figure S1: Kaplan–Meier survival validation of the intersected prognosis‐related pathways. Pathways were dichotomized into high‐ and low‐activity groups, and OS and PFS were compared using Kaplan–Meier curves. Figure S2: Determination of the optimal factorization rank for NMF subtyping. Cophenetic, dispersion, evar, residuals, RSS, silhouette, and sparseness metrics were evaluated across ranks to determine the optimal value. Figure S3: Spot annotation in spatial transcriptomics based on representative markers. (A) H&E image of the tissue section. (B) Unsupervised clustering of spatial spots. (C) Spatial expression patterns of representative markers (tumor: EPCAM; stromal/immune: DCN, LYZ; normal: SFTPC) for niche annotation. (D) UMAP of spatial spots colored by niche type (tumor, stromal and immune, normal). [file HUMU-2026-4471260-s001.docx]

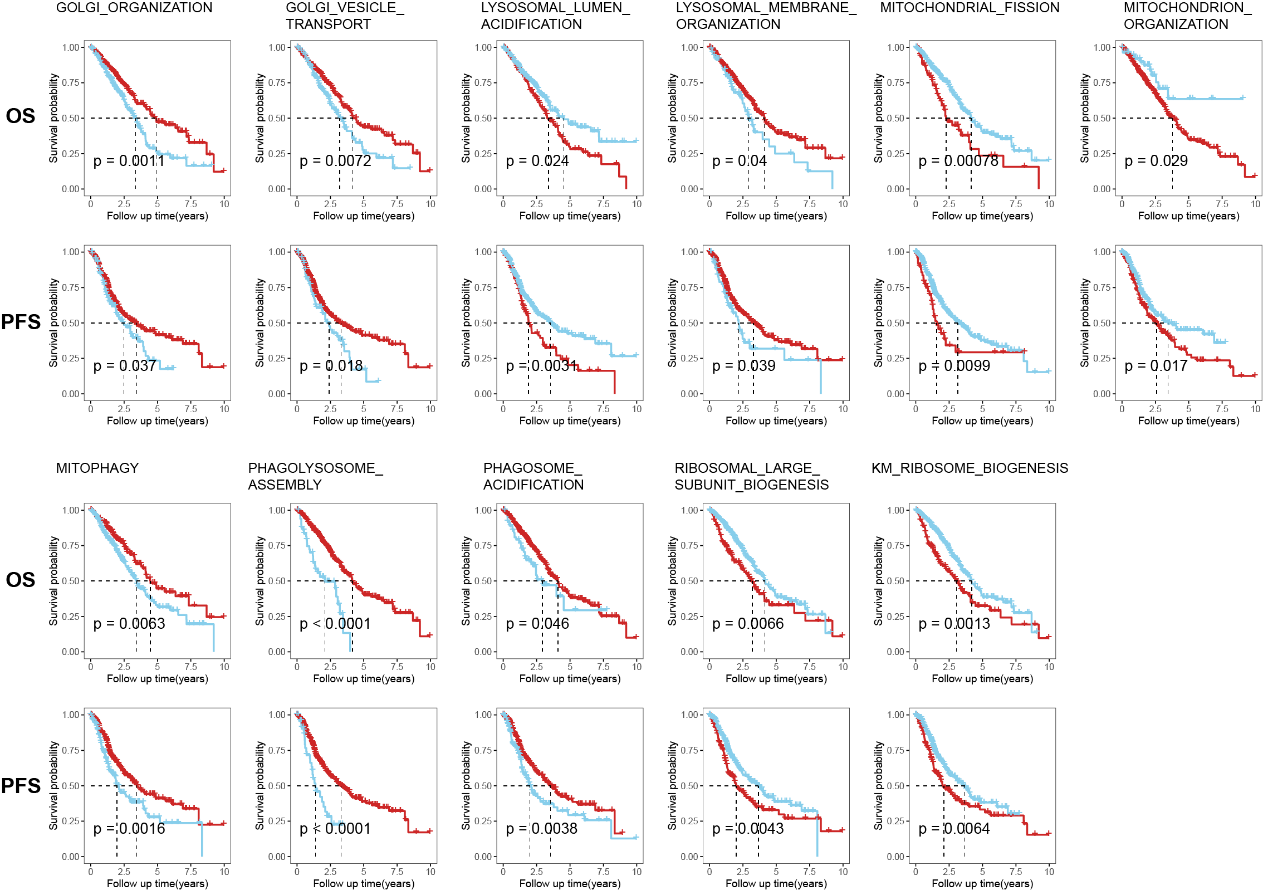


**Figure S1.** Kaplan–Meier survival validation of the intersected prognosis-related pathways. Pathways were dichotomized into high and low activity groups, and OS and PFS were compared using Kaplan–Meier curves.


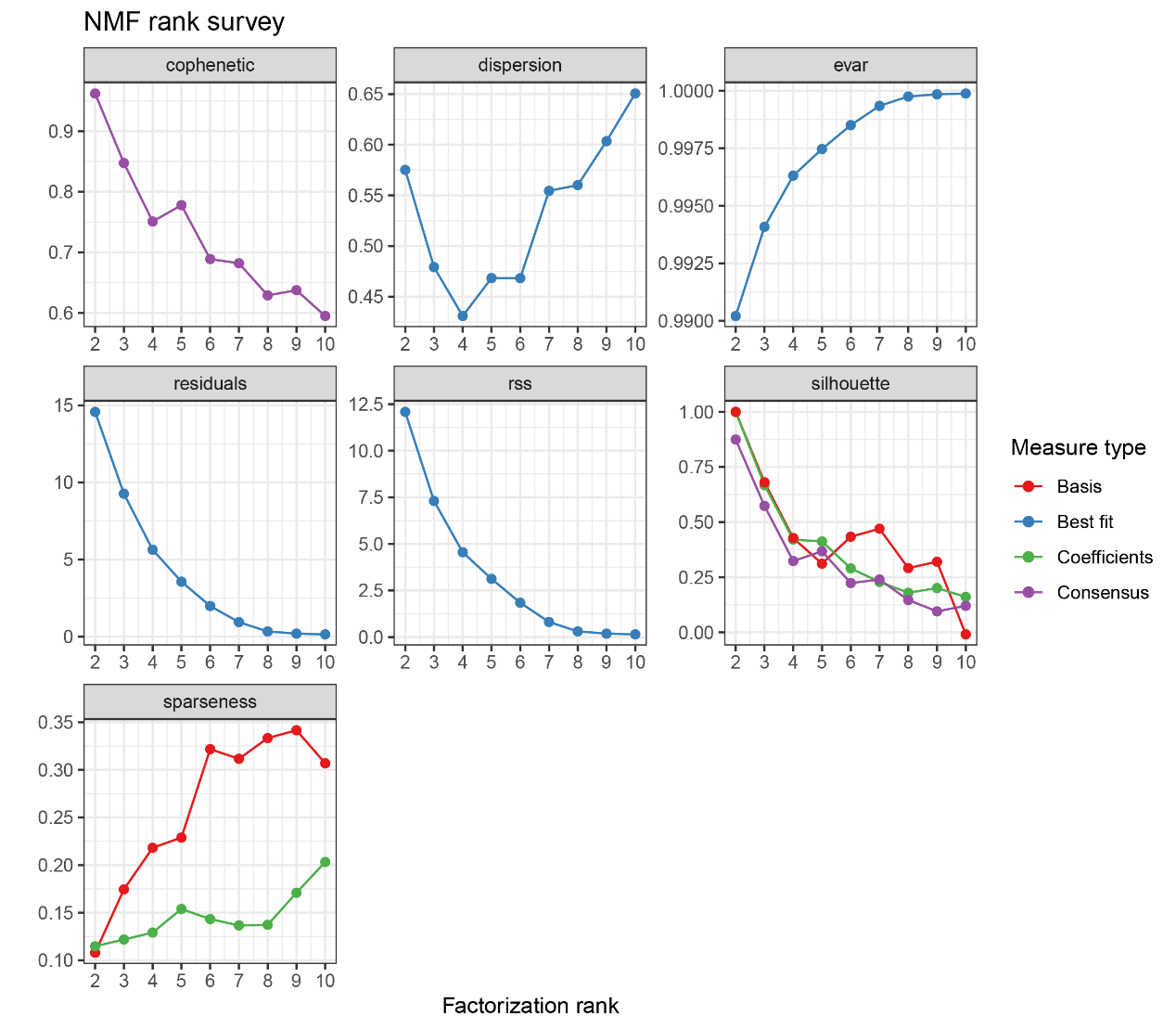


**Figure S2.** Determination of the optimal factorization rank for NMF subtyping. Cophenetic, dispersion, evar, residuals, RSS, silhouette, and sparseness metrics were evaluated across ranks to determine the optimal value.


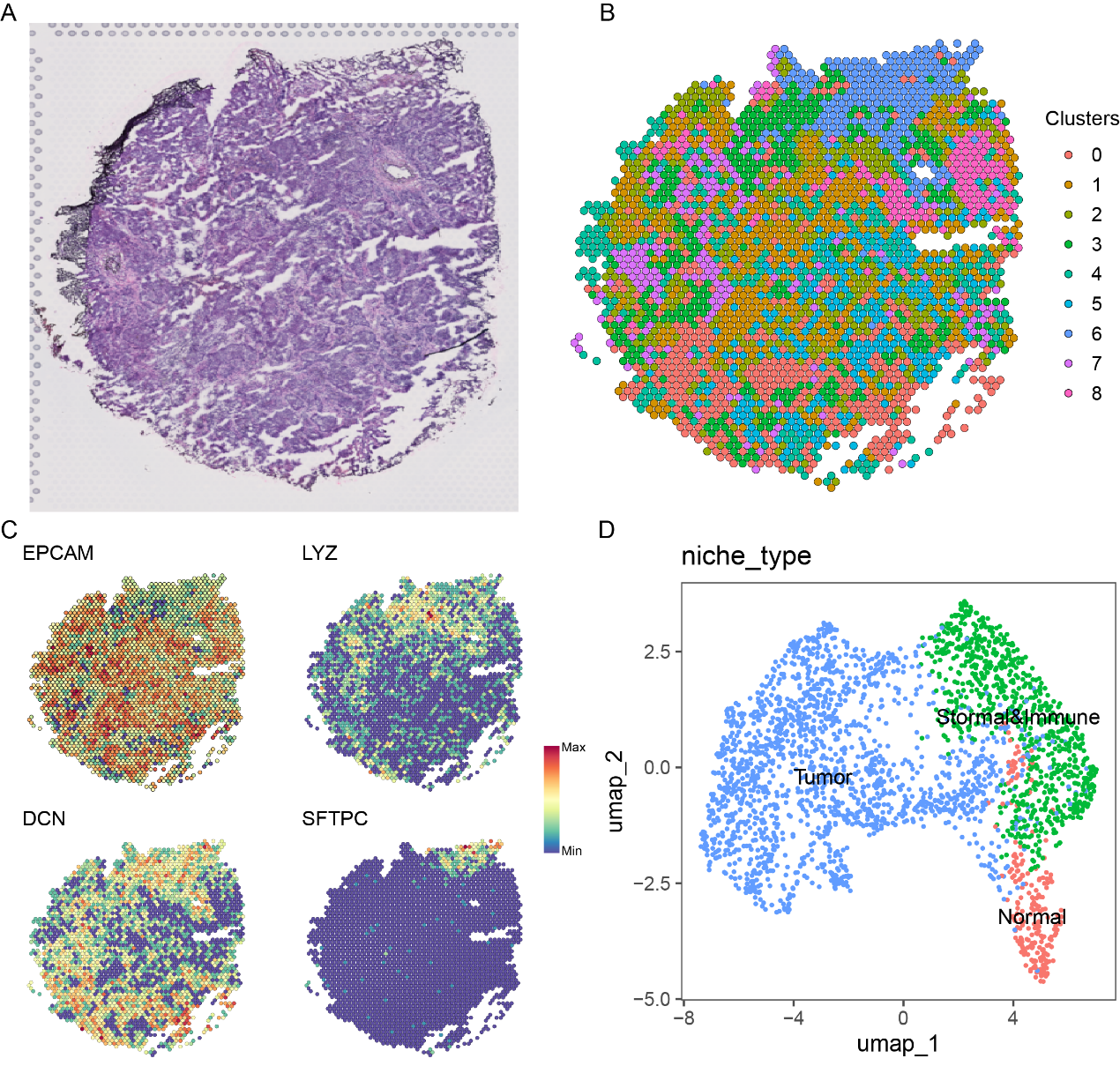


**Figure S3.** Spot annotation in spatial transcriptomics based on representative markers. (A) H&E image of the tissue section. (B) Unsupervised clustering of spatial spots. (C) Spatial expression patterns of representative markers (tumor: EPCAM; stromal/immune: DCN, LYZ; normal: SFTPC) for niche annotation. (D) UMAP of spatial spots colored by niche type (Tumor, Stromal and Immune, Normal).
